# Short animated video increases knowledge and perceived comfort in clinical counseling on inequitable health impacts of air pollution among interprofessional health learners and clinicians

**DOI:** 10.1186/s12909-023-04785-1

**Published:** 2023-11-12

**Authors:** Brenna M. Doheny, Jack J. Inglis, Karly A. Boll, Scott Lunos, Vishnu Laalitha Surapaneni

**Affiliations:** 1 Department of Family Medicine and Biobehavioral Health, University of Minnesota Medical School, Duluth campus, 1035 University Drive, Duluth, MN 55812-3031 USA; 2 Hennepin Healthcare, 701 Park Avenue, MN Minneapolis, 55415 USA; 3 Biostatistical Design and Analysis Center, Clinical and Translational Science Institute, University of Minnesota, Minneapolis, MN USA; 4 Department of General Internal Medicine, University of Minnesota Medical School, Minneapolis, MN USA

**Keywords:** Climate, Air pollution, Health equity, Clinical encounter, Climate change, Counseling, Climate change health impacts, Health and social inequities, Social justice, Air pollution, Environmental health, Health behavior, Health risk behavior, Professional education

## Abstract

**Background:**

Air pollution is a major health risk contributing to global morbidity and mortality, yet clinicians do not routinely engage in counseling patients on this topic. Clinicians cite their lack of education as a common barrier. We developed a two-minute animated video on mitigating air pollution health risks and evaluated the efficacy of this video as an educational tool.

**Methods:**

In March-June 2021, a convenience sample of Minnesota interprofessional health learners and clinicians viewed the video and completed an electronic survey that assessed pre-/post-video intervention changes in (a) didactic and clinically applied knowledge on health impacts of air pollution, (b) perceived comfort in identifying at-risk patients and counseling them on relevant preventive health behaviors, (c) intentions/barriers to counseling patients, (d) beliefs and attitudes related to the health harms of air pollution, and (e) perceptions of the overall acceptability of the intervention.

**Results:**

The 218 participants included learners and clinicians in medicine, nursing, and advanced practice provision. Respondents’ knowledge scores and self-reported level of comfort in identifying high-risk patients and counseling them on preventative health behaviors increased significantly pre-/post-intervention. The video also effectively altered participants’ misperceptions about the health impacts of air pollution. While less than half of participants (43.6%) reported they intended to engage in counseling patients as a result of watching the video, 52.3% indicated they might do so. Lack of time during clinical encounters and lack of training were reported as persistent barriers to engaging in this counseling. Overall, participants found the video to be an effective educational tool, indicating that they wanted their colleagues and patients to watch the video and would like to see further short, animated videos on other environmental health topics.

**Conclusions:**

A two-minute animated educational video significantly improved knowledge of inequitable health impacts of air pollution and improved perceived comfort in identifying and counseling at-risk patients among health professional learners and clinicians regardless of profession, level of training, or pre-intervention knowledge level. Academic health professional training programs and health systems should consider adopting this modality as a tool for educating learners, clinicians, and patients on environmental health risks.

**Supplementary Information:**

The online version contains supplementary material available at 10.1186/s12909-023-04785-1.

## Background

Air pollution is widely recognized as a significant public health risk with causal links to cardiovascular morbidity and mortality and new-onset childhood asthma [1–3], and leads to up to 200,000 deaths a year in the United States [4]. More than 4 in 10 Americans continue to live in areas with poor air quality [5]. Communities of color are disproportionately impacted [6, 7], with Black, Hispanic, and Asian communities exposed to higher levels of air pollution from particulate matter (PM2.5) regardless of their income level or state of residence. These inequitable health impacts should be recognized in the clinical environment and addressed through counseling on preventative behaviors. Yet, provider-patient counseling on mitigating the negative health impacts of this modifiable risk factor is not well documented in the literature. A 2018 study from the U.S. Centers for Disease Control and Prevention (CDC) found that only 3% of randomly selected respondents to a cross-sectional survey reported discussing air pollution with their healthcare providers, and among survey respondents who indicated they had heart disease, asthma, or emphysema/COPD, only 9% reported discussing air pollution with providers [8]. A further CDC study reported that less than half of healthcare providers surveyed ever discussed air pollution with their patients [9]. While interprofessional health students and clinicians recognize that environmental exposures and climate change are impacting the health of their patients [10–13], they cite a lack of education on these topics as a primary barrier to counseling patients [14, 15].

In published literature, several frameworks [16–18] and curricula have been developed for educating learners (defined for the scope of this paper to include medical students, residents, fellows, nursing students, and advanced practice provider students) [19–21] and clinicians (defined for the scope of this paper as physicians, nurses, and advanced practice providers) [22] on health impacts of environmental factors in a didactic setting. However, only a few studies have evaluated curricula that specifically address how learners and clinicians should approach a clinical encounter with an at-risk patient [23–26].

This gap in the literature, of designing and evaluating curricula that effectively teach clinical counseling skills, is critical and prompts a need for further study. This is because in addition to long-term health impacts, poor air quality is related to real-time health impacts. For example, there is a rise in emergency room visits and hospitalizations related to respiratory issues [27, 28], atrial fibrillation [29], and myocardial infarction [30] on days with high air pollution. Theoretically, counseling on air pollution can decrease this burden. Indeed, patients report valuing health professional counseling on environmental factor impacts, and health professional counseling has been shown to increase patient likelihood to engage in recommended behaviors (e.g., climate change mitigation behaviors such as reducing energy use) [31].

While designing our study intervention, we reviewed the literature for barriers of uptake of environmental health education in medicine. The perception that climate-health education would add to the ever-increasing scope of social determinants of health in which health professionals are supposed to be well-versed [32–34] and the lack of faculty with local expertise in this relatively new and rapidly evolving field [32, 35, 36] are cited, highlighting the need to create and evaluate curricular components that are brief and can be self-administered asynchronously by learners and clinicians at their convenience, such as on their mobile devices. Videos, especially those with animation [37, 38], are increasingly considered quick and effective educational tools [39], as stand-alone resources or to enhance multimodal learning in interdisciplinary graduate health education [40, 41]. Thus, we designed a two-minute animated video that describes how to identify and counsel patients at risk of health impacts of air pollution.

In this study, we evaluated the efficacy of the video as a tool to promote positive changes in the following outcomes for health professionals: (a) knowledge of health impacts of air pollution and its clinical application, (b) perceived comfort in identifying patients at risk of health harms of air pollution and counseling them on relevant preventive health behaviors, (c) intentions and barriers to counseling patients on health harms of air pollution, (d) beliefs and attitudes related to health harms of air pollution, and (e) perceptions of the overall acceptability of the intervention.

## Methods

### Study design, setting, and participants

We designed this study as a pre/post intervention to assess changes in the knowledge, perceived comfort, and beliefs and attitudes of health professional trainees and clinicians after viewing a brief educational video on the health effects of air pollution. From March through June 2021, we recruited a convenience sample of interprofessional health learners (students, residents, fellows) and practicing clinicians (nurses, physicians, and advanced practice providers) across Minnesota by asking the administrative leaders of medical departments (e.g., Internal Medicine, Family Medicine, Pediatrics) in three health systems in the state, the University of Minnesota (UMN) School of Nursing, UMN School of Medicine, a Physician Assistant school, and a local medical society (American College of Physicians, Minnesota chapter) to share an anonymous link to our study materials in emails and newsletters sent to their members. After completing a pre-intervention survey, participants watched the two-minute educational video linked in the survey platform, then completed a post-intervention survey. As the pre-intervention survey occurred directly prior to the video intervention, and the post-intervention survey directly after, no other education the participants may have received could have impacted the findings.

After submitting a completed survey, participants were offered a $10 gift card incentive. The UMN Institutional Review Board determined the study to be exempt from review.

### Video development

VLS, an internal medicine physician with over 5 years of experience leading climate health education in academic, clinical, and public outreach settings, conceptualized the video and wrote the script with JJI and KAB, and we contracted with Kindea Labs to design and produce the animated video. Using the Flesch-Kincaid scale [42], the script had an eighth-grade level readability. The video and its complete transcript are available as Additional File 1 and Additional File 2, respectively.

The video is set up as a clinical encounter. The physician in the video describes the health impacts of air pollution and the current state of inequitably distributed burden of disease in Minnesota’s low-income and Black, Indigenous, and People of Color (BIPOC) communities, drawing from data from the Minnesota Pollution Control Agency [7]. Next, using the vulnerability assessment for sustainability framework [43], the physician explains how to identify patients who are at risk for air pollution-related health impacts, outlining that those with high exposure and high sensitivity and those with the least adaptive capacity are at highest risk. The video ends with a description of preventative behaviors patients can adopt to minimize the impact of air pollution on their health. Three practicing clinicians from across the state, a family medicine physician with experience in environmental health, a health psychologist engaged in climate justice research, and a pulmonologist, reviewed the script for accuracy and quality testing.

### Survey methods and outcome measures

VLS, JJI, and KAB developed the survey instrument. The pre-intervention survey collected demographics and information about participants’ previous training in health impacts of environmental factors and in identifying at-risk patients. The pre/post survey included questions related to our educational outcomes of interest: (a) didactic knowledge of health impacts of air pollution and clinically applied knowledge in identifying at-risk patients, (b) comfort in identifying and counseling at-risk patients, (c) intentions and barriers to counseling patients on health harms of air pollution, and (d) attitudes and beliefs related to the health harms of air pollution; the post-intervention survey included additional questions assessing outcome e), participants’ perceptions of the educational video and its utility as a learning tool. The complete survey, with correct answers to knowledge questions indicated, is available as Additional File 3.

The majority of the survey questions were closed-ended. We assessed changes in participant knowledge over 12 multiple choice or true/false questions, and calculated a composite score as the sum of correct answers (where each correct answer was assigned a score of 1, and each incorrect answer or “unsure” was assigned a score of 0). Participants rated their comfort in identifying and counseling patients on a five-point Likert scale (from extremely uncomfortable to extremely comfortable), and indicated their attitudes and beliefs related to the health harms of air pollution on a seven-point Likert scale (from strongly disagree to strongly agree). We collected information on intentions and barriers to counseling patients and perceptions on the video as a training tool using multiple choice questions. In addition, the post-intervention survey included optional open-ended questions asking participants what surprised them most about the video, and for any further feedback on the video or survey itself.

We developed and administered the survey instrument in the QualtricsXM platform (Qualtrics, Provo, UT). Two medical students, one nursing student, and two practicing internal medicine physicians tested the survey for technical functionality and clarity of questions before it was sent to participants.

### Data analysis

The data were analyzed using SAS V9.4 (SAS Institute Inc., Cary, NC). We used descriptive statistics (counts and percentages for categorical variables; means, medians, and standard deviations for continuous/ordinal variables) to summarize data on participants’ demographic characteristics and their self-reported level of prior training in the health impacts of environmental factors and in methods for identifying and counseling at-risk patients. To measure post-intervention changes in knowledge, comfort in identifying and counseling at-risk patients, and attitudes related to health harms of air pollution, we conducted paired t-tests for scaled responses and McNemar’s tests for categorical responses. With a target sample size of 250, a paired t-test would have 80% power to detect an effect size of 0.18 using a 0.05 level of significance.

To determine whether there were any differences among demographic categories in the mean change in composite knowledge scores, perceived comfort in identifying and counseling at-risk patients, and changes in beliefs and attitudes, we conducted analysis of variance (ANOVA), and post-hoc Tukey pairwise comparisons when the overall ANOVA was significant (*P* < 0.05). This method was also utilized to explore associations between pre-intervention interest and post-intervention intention to counsel patients on air pollution and health. Pearson correlation was used to determine if there was a relationship between participants’ pre-intervention level of knowledge and their post-intervention change in comfort in counseling patients.

To analyze responses to the open-ended question querying what surprised participants most about the video, a member of the research team trained in qualitative analysis (BD) inductively developed a thematic coding framework and coded the responses. To assess the relative importance of each theme, results were quantitated by counting the frequency of each theme, and percentages were calculated as the frequency of a given category or subcategory out of the total number of valid responses.

## Results

### Participants

Demographic characteristics of the 218 participants who completed the survey are summarized in Table 1. The majority were learners and clinicians in medicine (MD/DO; 75.2%), cis-gender female (68.8%), white (82.1%), and in urban practice settings (79.4%). A total of 3.7% of respondents were students or clinicians in nursing (RN), and 19.2% were advanced practice providers (APPs) or APP students.

**Table 1:** Demographic characteristics of survey respondents (*n* = 218)

Demographic characteristic	Category	n (%)
Profession^a^	Medicine (MD/DO)	164 (75.2)
	Nursing (RN)	8 (3.7)
	Advanced Practice Provider (APP)^b^	42 (19.2)
	Other^c^	4 (2.3)
MD/DO Level of training/practice	Medical Student	53 (32.5)
	Residents/Fellows	47 (28.8)
	0–10 yrs. in practice	29 (17.8)
	> 10 yrs. in practice	34 (20.9)
Gender Identity	Cis-gender Female	150 (68.8)
	Cis-gender Male	63 (28.9)
	Non-binary	1 (0.5)
	Prefer not to answer	4 (1.8)
Ethnic/Racial Background (respondents may pick more than 1)	Asian or Asian Indian	21 (9.6)
	Black or African American	3 (1.4)
	Caucasian/White	179 (82.1)
	Hispanic, Latino, or Spanish	3 (1.4)
	Middle Eastern or Northern African	4 (1.8)
	Multiracial	3 (1.4)
	Native American or Alaska Native	5 (2.3)
	Other	2 (1.0)
	Prefer not to answer	7 (3.2)
Specialty	Internal Medicine	54 (24.8)
	Family Medicine	42 (19.3)
	Pediatrics	30 (13.8)
	Other	44 (20.2)
	N/A (medical students)	48 (22.2)
Practice Location (respondents may pick more than 1)	Urban	173 (79.4)
	Suburban	42 (19.3)
	Rural	34 (15.6)
	Tribal	5 (2.3)
Practice Setting	Inpatient	49 (22.5)
	Outpatient	41 (18.8)
	Both	102 (46.8)
	Other	6 (2.4)
	Non-patient facing	21 (9.6)
Political Leaning	Conservative	14 (6.4)
	Liberal	140 (64.2)
	None/Varies	49 (22.5)
	Other	3 (1.4)
	Prefer Not to Say	12 (5.5)

^a^Profession queried as degree pursuing/obtained

^b^Advanced Practice Providers (APPs) include Physician Assistants (PA) and Nurse Practitioners (NP)

^c^“Other” includes BA ( 1), CMA ( 2), PhD ( 1)

### Knowledge

Prior to viewing the video, 85.3% of respondents (186/218) indicated receiving “a little” or “none at all” training or education on the health impacts of environmental factors such as climate change and air pollution. From pre- to post-intervention, there was a significant increase in scores for 7 of the 12 knowledge questions and for the overall composite score (from 7.7 to 9.7, *P* < 0.001; Table 2).

**Table 2:** Pre/Post intervention didactic and clinically applicable knowledge

	Pre-intervention n (%)	Post-intervention n (%)	*P*-value^a^
**Didactic Knowledge**			
Air pollution has been shown to be associated with the following health impacts:			
Increased risk of stroke			< 0.0001
Yes	82 (37.6)	193 (88.5)	
No	8 (3.7)	7 (3.2)	
Maybe	128 (58.7)	18 (8.3)	
Increased risk of heart attack			< 0.0001
Yes	123 (56.4)	201 (92.2)	
No	7 (3.2)	5 (2.3)	
Maybe	88 (40.4)	12 (5.5)	
Increased risk of acute asthma exacerbation			0.3173
Yes	215 (98.6)	217 (99.5)	
No	1 (0.5)	1 (0.5)	
Maybe	2 (0.9)	0	
Increased risk of acute exacerbation of chronic obstructive pulmonary disease (COPD)			0.0047
Yes	215 (98.6)	203 (93.1)	
No	0	8 (3.7)	
Maybe	3 (1.4)	7 (3.2)	
Increased risk of type 2 diabetes			0.0005
Yes	39 (17.9)	63 (28.9)	
No	37 (17.0)	93 (42.7)	
Maybe	142 (65.1)	62 (28.4)	
Increased risk of dementia			0.1086
Yes	58 (26.6)	69 (31.6)	
No	14 (6.4)	85 (39.0)	
Maybe	146 (67.0)	64 (29.4)	
Approximately how many deaths per year does air pollution contribute to in Minnesota?			< 0.0001
Correct	56 (25.7)	194 (89.0)	
Incorrect	162 (74.3)	24 (11.0)	
**Clinically applied knowledge**			
Of the following, which groups are the most sensitive to air pollution in Minnesota?			0.0006
Correct	188 (86.2)	208 (95.4)	
Incorrect	6 (2.8)	9 (4.1)	
Unsure	24 (11.0)	1 (0.5)	
Of the following, which groups are most likely to have high exposure to air pollution in Minnesota?			0.0029
Correct	194 (89.0)	207 (95.0)	
Incorrect	6 (2.8)	7 (3.2)	
Unsure	18 (8.3)	4 (1.8)	
Which group is least likely to have adaptive capacity (ability to protect themselves) regarding air pollution impacts?			0.6831
Correct	194 (89.0)	196 (89.9)	
Incorrect	10 (4.6)	19 (8.7)	
Unsure	14 (6.4)	3 (1.4)	
Which of the following Minnesotans is at lowest risk for experiencing health consequences related to air pollution?			0.4233
Correct	173 (79.4)	178 (81.6)	
Incorrect	26 (11.9)	30 (13.8)	
Unsure	19 (8.7)	10 (4.6)	
On days with poor air quality, what is the best way to limit harm from outdoor air pollution?			< 0.0001
Correct	137 (62.8)	176 (80.7)	
Incorrect	42 (19.3)	31 (14.2)	
Unsure	39 (17.9)	11 (5.1)	
**Composite Score** (0–12), mean (SD) [median]			
	7.7 (2.0) [8.0]	9.7 (1.6) [10.0]	< 0.0001

^a^McNemar’s test comparing proportion correct between pre- and post-video. Paired t-test was used to compare mean composite score between pre- and post-video. Composite score is the total correct of the 12 knowledge questions (yes = 1, maybe = 0, and no = 0)

The mean change in pre/post-intervention composite knowledge score did not differ significantly among the three professional groups or among the four training categories of medical professionals. Sample sizes of APP and RN respondents were too low to compare among training categories.

### Perceived comfort in patient counseling

Pre-intervention, 89.0% (194/218) of respondents reported having received “a little” or “none at all” training or education on counseling patients at risk of negative health impacts of environmental factors such as air pollution or climate change. From pre- to post-intervention, there was a significant increase in comfort in identifying patients who would be at high risk for health impacts of air pollution and in counseling patients on preventative health behaviors (Table 3). There were no differences in mean pre-/post-intervention score change among professions or medical professional training categories.

**Table 3:** Pre/Post intervention perceived comfort in counseling patients on health harms of air pollution

	Pre-intervention mean (SD) [median]	Post-intervention mean (SD) [median]	Difference mean (SD) [median]	*P*-value^a^
Describing the health impacts of air pollution in an academic setting (e.g., when discussing a patient on rounds)	2.6 (1.1) [2.5]	3.3 (1.0) [4.0]	0.72 (1.11) [1.0]	< 0.0001
Identifying which patients are high-risk for health impacts secondary to air pollution in a clinical setting	3.1 (1.0) [3.0]	3.8 (0.9) [4.0]	0.71 (1.09) [1.0]	< 0.0001
Counseling patients on their personalized risk for negative health impacts secondary to air pollution	2.7 (1.0) [3.0]	3.6 (0.9) [4.0]	0.89 (1.12) [1.0]	< 0.0001
Counseling patients about health behaviors to protect themselves from the risks of air pollution	2.7 (1.0) [2.0]	3.6 (0.9) [4.0]	0.97 (1.15) [1.0]	< 0.0001

^a^*p*-values are from paired t-tests. Mean (SD) [median] are presented in the table. Likert scale ranged from 1 = extremely uncomfortable to 5 = extremely comfortable

There was no correlation between pre-intervention knowledge scores and change in comfort at identifying at-risk patients (*r* = -0.09, *P* = 0.64), counseling patients on their personalized health risks (*r* = -0.04, *P* = 0.56), or counseling patients on preventative health behaviors (*r* = -0.06, *P* = 0.40).

### Counseling intentions and barriers

Prior to watching the video, over half of the respondents (125/218, 57.3%) indicated that they never engage in counseling patients on the effects of air pollution. When queried whether they intend to identify and counsel patients at risk of adverse health impacts of air pollution as a result of watching the video, 43.6% (95/218) answered “yes” and 52.3% (114/218) “maybe”. Nearly a third (38/125, 30.4%) of those who had never counseled patients before indicated that they would do so as a result of watching the video. Post-intervention intent was significantly correlated with respondent agreement level (on a five-point Likert scale) with the statement “I have wanted to counsel my patients on air pollution and health but have not done so due to lack of training/knowledge” on the pre-intervention survey (ANOVA, *P* < 0.0001). Post-hoc Tukey pairwise comparisons indicated that those who responded “yes” had a higher mean level of agreement [4.5 (SD 1.3)] when compared to those who answered “maybe” [3.8 (SD 1.4), *P* < 0.001], or “no” [3.1 (SD 2.5), *P* = 0.01].

When evaluating persistent barriers to counseling patients, of the 123 responding participants, 60.2% (*n* = 74) reported limited time in a clinical encounter and 57.7% (*n* = 71) reported a need for more training.

### Beliefs and attitudes regarding health impacts of air pollution

From pre- to post-intervention, there were significant changes in 5 of the 6 beliefs regarding the health impacts of air pollution (Table 4). There was significantly increased agreement that air pollution currently impacts the health of patients, including patients in rural areas; that air pollution has inequitable health impacts; and that climate change will contribute to worsening air quality. There was significantly decreased agreement with the misperception that air quality in Minnesota is good enough that it does not impact health.

**Table 4:** Pre/Post intervention beliefs and attitudes on health harms of air pollution

	Pre-intervention mean (SD) [median]	Post-intervention mean (SD) [median]	Difference mean (SD) [median]	*P*-value^a^
For the following questions, please select the extent to which you agree or disagree with the statement:				
Air pollution negatively affects human health	6.4 (1.1) [7.0]	6.5 (0.9) [7.0]	0.07 (1.12) [0.0]	0.3652
Air quality in Minnesota is good enough that it does not have significant health impacts on Minnesotans	3.7 (1.5) [4.0]	2.3 (1.4) [2.0]	-1.37 (1.72) [-1.0]	< 0.0001
Air pollution impacts the health of patients that I currently care for in Minnesota	5.1 (1.4) [5.0]	6.1 (1.0) [6.0]	1.08 (1.45) [1.0]	< 0.0001
Air pollution has a significant impact on the health of Minnesotans who live in rural areas	4.5 (1.5) [5.0]	5.7 (1.2) [6.0]	1.18 (1.49) [1.0]	< 0.0001
Some Minnesotans are more negatively affected by air pollution than others	6.3 (0.9) [7.0]	6.6 (0.9) [7.0]	0.26 (1.11) [0.0]	0.0008
Climate change will contribute to worsening air quality over the coming decades	6.2 (1.1) [7.0]	6.5 (0.9) [7.0]	0.25 (0.81) [0.0]	< 0.0001

^a^*p*-values are from paired t-tests. Mean (SD) [median] are presented in the table. Likert scale ranged from 1 = strongly disagree to 7 = strongly agree

There were no significant differences in the change in pre/post-intervention scores among professions or among levels of training of medical professionals.

A total of 213 participants responded to the open-ended question “What surprised you most about the information that was presented in the video?” We identified ten themes among the responses (Table 5), the most common being surprise at the number of deaths associated with air pollution in Minnesota.

**Table 5:** Thematic analysis of open-ended responses to video

Theme	Subtheme	Frequency	Percentage	Example
Number of deaths		74	34.7	Higher number of Minnesotans die of it than I thought.
non-respiratory health impacts		33	15.5	How many diseases it can cause/exacerbate that are not respiratory related
	stroke	29	13.6	I didn’t realize it contributed to increased incidence of stroke
	heart disease	16	7.5	The link to heart disease
	diabetes	1	0.5	Diabetes and stroke were higher risks of air pollution
rural Minnesota affected		31	14.6	How the poor air quality spread into the rural communities far away from the metro area
children more susceptible		24	11.3	Children are more exposed to pollutants because of their increase in respiration
nothing		23	10.8	Unfortunately I wasn’t surprised
Overall extent of air pollution in MN		22	10.3	How widespread the impact is in MN, I thought it was more of a CA thing
critical of video/survey		5	2.3	That we are not talking more about prevention rather than ‘adaptation’. Industrial farming, pesticides, vehicle exhaust, factory/industry pollutants are all contributors to air pollution, and it seems too far down stream to be counseling patients about adaptive capacity when the problem will only worsen without upstream interventions/policy/accountability.
actions that can be taken		4	1.9	That we shouldn’t go outside if the air quality is bad
adaptive capacity/exposure parameters		2	0.9	I was not aware of the different components of exposure parameters
disproportionate impacts on Indigenous people		2	0.9	How disparately American Indians are affected

Qualitative analysis of responses from Minnesota-based interprofessional health learners and clinicians to the open-ended question, “What surprised you most about the information that was presented in the video?” (*n* = 213)

### Perceptions of the video as an educational tool

The response to questions evaluating the acceptability of the video was overwhelmingly positive. A total of 91.2% (198/217) strongly or somewhat agreed that they learned useful information from the video, 96.3% (210/218) felt the information was easy to understand, and 84.9% (185/218) wanted to watch similar videos on other topics related to environmental health. A total of 34.9% (76/218) felt it was too simple or left out valuable information.

Nearly three-quarters of respondents (72.9%; 159/218) strongly or somewhat agreed that they would want their colleagues to watch this video, and 94.0% (205/218) wanted their patients to watch this video. Out of a list of scenarios for showing the video to patients, 70.7% (145/205) chose “waiting room”, 61.5% (126/205) viewing via electronic medium (e.g., MyChart message), and 10.2% (21/205) chose “during clinical visit with the provider”.

The vast majority of learners (88.2%, 97/110) agreed that they would like their academic program to provide clinical training on counseling patients on risks of air pollution and protective behaviors. When these learners were asked to select from a list of preferred modalities for this training, 73.2% (71/97) preferred animated videos such as the one in the study, 67.0% (65/97) recorded lectures, 29.9% (29/97) in-person lectures, and 27.8% (27/97) reading materials.

## Discussion

Our self-administered two-minute animated video resulted in significant increases in knowledge and positive changes in beliefs and attitudes regarding the health impacts of air pollution among Minnesota-based interprofessional health learners and clinicians. This intervention also significantly increased participants’ perceived comfort in identifying and counseling at-risk patients on their personalized health risks of air pollution and health protective behaviors. To our knowledge, this is the first study to use this type of short educational video intervention and report significant gains in clinician knowledge, comfort, and attitudes around environmental health.

Other interventions that have shown efficacy have involved longer timeframes and more complex and intensive logistics. For example, a voluntary eight-week online course for an interdisciplinary health professional audience showed an increase in confidence and skills counseling patients [26]. A 6-week environmental health module with didactic and experiential elements at New Jersey Medical School improved the confidence of medical students in counseling their patients [25]. University of Illinois Chicago Medical School found the standardized patient encounter format to be an effective teaching technique [24]. In Spain, augmented reality was used over a three-year period to teach nurses how to care for a patient during a heatwave as well as about sustainability measures in healthcare [23].

The majority of our participants reported that they had not previously received instruction on health impacts of air pollution or training on counseling patients on their personalized environmental health risks and health protective behaviors, underscoring the importance of integrating environmental health into medical education. The pre-intervention data showed that while there was good understanding of respiratory health impacts (asthma and COPD exacerbation), there was a gap in knowledge of the wide-ranging health impacts of air pollution, including its effects on ischemic heart disease and stroke [44], cardiovascular mortality [45], diabetes [46], and dementia [47]. Given the well-established causal relationship between air pollution and cardiovascular mortality, there is a critical public health need for educating health professionals in this topic. Our video intervention resulted in a significant increase in didactic knowledge of topics that were cited in the video, including cardiovascular impacts of air pollution as well as the burden of disease in Minnesota. In contrast, knowledge of asthma exacerbations did not significantly increase post-intervention, likely because pre-intervention knowledge was already high. Identification of COPD exacerbation as an impact of air pollution decreased significantly post-intervention, but this is likely because COPD was not specifically referenced in the video.

Participants did not see a uniform increase in clinically applied knowledge after watching the video. While there were significant increases in participants’ ability to identify those patients most sensitive and at highest exposure risk, ability to identify those with lowest adaptive capacity and those at lowest risk of health consequences did not increase significantly. These concepts are difficult to assess in a survey questionnaire format, as context is required to make such determinations. It is likely that more nuanced instruction is necessary to understand how various risk factors of an individual patient interact with air pollution exposure to result in adverse health outcomes. Overall, the mean composite knowledge score significantly increased after the intervention, and increased knowledge can help health professionals identify patients that would benefit from counseling. Moreover, the increase in knowledge occurred across all professions and training levels, indicating that this intervention has broad efficacy.

After the video intervention, all participants had increased levels of perceived comfort in identifying at-risk patients and educating and counseling them on health protective behaviors. While pre-intervention knowledge was positively associated with pre-intervention comfort levels, there was no correlation between change in comfort level and pre-intervention knowledge scores, indicating that the video intervention was effective in increasing participants’ comfort levels independent of their level of prior knowledge.

This study revealed commonly held misconceptions about the health impacts of air pollution among participants, and that the video intervention was effective in altering them. Both the knowledge assessment and responses to the open-ended question regarding what was most surprising about the video indicated that participants believed that air pollution was not a significant problem in Minnesota, and that rural populations are not impacted by air pollution. The video intervention significantly changed these perceptions, as indicated by increased agreement scores, suggesting that short, evidence-based information from reliable sources is effective in changing attitudes and beliefs among healthcare professionals, which may then also impact their practice behaviors. These increases are especially notable in the context of self-selecting participants opting into an educational intervention related to environmental health.

Although the video intervention significantly increased participant knowledge and comfort in counseling patients and reduced misconceptions regarding environmental health impacts, less than half of participants reported definite intent to identify and counsel patients at risk of adverse health impacts as a result of watching the video. However, very few respondents (4.1%) stated outright that they would not engage in counseling, and the majority indicated they would “maybe” do so. Additionally, 40% of respondents who indicated definite intent to counsel at-risk patients as a result of watching the video had never engaged in counseling patients on air pollution before. Taken together, this suggests the video had some efficacy in motivating providers to directly take action, but that they may experience barriers that limit their ability to act.

One of the most commonly cited barriers to counseling patients in both this study and others [15] was lack of time during the clinical encounter. A study analyzing records of primary care visits from 1998 to 2000 found the average duration of a clinical encounter was 17.4 min [48], and despite the increased medical complexity of patients, and increased number of quality measures physicians are now expected to cover compared to 20 years ago, the average duration of a primary care visit based on 2017 data was only 18 min [49]. This time pressure is a barrier that is indicative of larger issues in healthcare that deprioritize preventive health, including environmental health, and a multi-disciplinary, system-wide approach is needed to actualize much-needed change.

One potential time-saving approach for bridging the gap in patient education on air pollution could be incorporating short videos vetted by providers, presented in the waiting room during clinical visits or via electronic medical record messages. Animated patient education videos are increasingly used for a variety of topics [50–52], and a recent meta-analysis of studies evaluating the efficacy of such videos found an overall positive effect in improving patient knowledge across health topics and clinical contexts [53]. The majority of participants in our study indicated they wanted their patients to watch the video in this intervention. The utility of this should be investigated further, and if found effective, this model could be expanded to a series of short videos alerting patients on how to protect their health in a time-sensitive manner during extreme heat waves, wildfire smoke events, or other extreme weather events that are predicted to become more common and severe with worsening global climate change.

A second commonly cited barrier to engaging in patient counseling about the health impacts of air pollution was the need for more training. The majority of learners in this study wanted their academic program to provide such training, underscoring the critical need to integrate environmental health into curricula. Given the ever-increasing demands on medical education, these findings support the development of a series of short, self-administered videos addressing geographically specific environmental health impacts as an effective educational tool. Self-administered videos can be utilized as stand-alone components, or augment existing curricula to reduce instruction time, and participants indicated this as a preferred modality over reading materials or in-person lectures.

Our study has several limitations. Our survey results were collected as a convenience sample, which increases the concern of sampling bias to those who are highly interested in issues of air pollution and environmental health. Although we made efforts to recruit an interprofessional sample, a higher proportion of physicians and medical trainees than other groups responded to the survey. The small sample size of nurses in particular makes it difficult to draw conclusions about the effectiveness of the intervention in that professional group, as well as its utility for interprofessional training. A higher proportion of our sample identified as white and as cisgender women than is reflected in statewide demographic data for medical students (62.4% women, 63.1% white per unpublished date from the University of Minnesota Medical School Office of Admissions) and for currently practicing physicians (35.6% women, 76.1% white) [54]. These demographic differences may limit the generalizability of our findings. While participants demonstrated increased intent to counsel in the immediate post-intervention period, the lack of follow-up makes it difficult to accurately predict actual change in practice.

For our next steps, we plan to test the proposed utility of this short, animated video for direct patient education via health professional guided dissemination. We will also continue to study video as a mode of educating health professional learners and clinicians on impacts of environmental health risks, including extreme heat. With much of the world facing health impacts of global climate change today, this is an area of great public health import.

## Conclusions

We found that a two-minute animated educational video significantly improved didactic knowledge of health impacts of air pollution, changed attitudes and beliefs, and improved confidence in identifying and counseling at-risk patients among health professional students and practitioners regardless of profession, level of training, or pre-intervention knowledge level. Short, animated videos show promise as a potentially effective tool for educating learners and clinicians as part of health professional curricula, paving the way for studying their effectiveness in directly educating patients on personalized environmental health risks.

### Supplementary Information

**Additional file 1.** The animated educational video evaluated in this study.

**Additional file 2.** Full transcript of the animated educational video evaluated in this study.

**Additional file 3.** Complete survey instrument administered to participants in this study, with correct answers to knowledge questions indicated.

## Data Availability

The datasets used and/or analyzed during the current study are available from the corresponding author on reasonable request.
