# Supplementary material for: Short animated video increases knowledge and perceived comfort in clinical counseling on inequitable health impacts of air pollution among interprofessional health learners and clinicians
Source: BMC Med Educ. 2023 Nov 12;23:858. doi: 10.1186/s12909-023-04785-1 (PMC10642052; doi:10.1186/s12909-023-04785-1)
Supplement: Supplementary file 2 — Additional file 2. Full transcript of the animated educational video evaluated in this study. [file 12909_2023_4785_MOESM2_ESM.docx]

**Additional File 2. Full transcript of the animated educational video evaluated in this study.**

**Jogger:** Like most Minnesotans, I love spending time outdoors. But I saw a report from our pollution control agency that said air pollution contributed to the deaths of 2000 to 4000 Minnesotans in a single year. Am I at risk?

**Doctor (female)**: Even though Minnesota has good air quality overall, we are still seeing health effects from air pollution. And climate change is predicted to further worsen our air quality in the coming decades. However, not everyone is affected the same by air pollution. Doctors consider three different factors to know who is at risk and how to reduce that risk.

Vehicles and other mobile equipment account for about half of overall air pollution in our state. Exhaust from vehicles has harmful pollutants. These pollutants can flare up asthma and cause heart attacks and strokes. People living near busy streets and highways are exposed to more pollution. And pollution isn’t just a city problem. Rural Minnesotans are getting hurt by air pollution too.

In our state, people who live in low-income communities, Indigenous communities and Communities of color suffered the worst impacts of air pollution.

Certain groups of people are more at risk to the harms of air pollution than others. For example, children breathe more number of times a minute and spend more time outdoors. So, they take in more air pollution.

Let’s say you have asthma or heart disease, the same level of air pollution hurts your health more than someone who is healthy.

The risks from air pollution can be decreased by taking steps to protect yourself or by improving your “adaptive capacity”.

There are several things you can do to protect yourself and your family from the harms of air pollution

- Check the air quality at “Air Now” before you go outside. If the air quality is poor, stay indoors if possible.
- If you have asthma, carry your inhaler with you at all times.
- Make an asthma action plan with your doctor.

Talk to your doctor about how you can protect your health from air pollution.
