# Supplementary material for: Short animated video increases knowledge and perceived comfort in clinical counseling on inequitable health impacts of air pollution among interprofessional health learners and clinicians
Source: BMC Med Educ. 2023 Nov 12;23:858. doi: 10.1186/s12909-023-04785-1 (PMC10642052; doi:10.1186/s12909-023-04785-1)
Supplement: Supplementary file 3 — Additional file 3. Complete survey instrument administered to participants in this study, with correct answers to knowledge questions indicated. [file 12909_2023_4785_MOESM3_ESM.docx]

**Additional File 3. Complete survey instrument administered to participants in this study, with correct answers to knowledge questions indicated. pdf**

**MN Air Pollution and Health Survey**

**Health Impacts of Air Pollution:**
**Evaluation of Current Training and Clinical Practice**

You are invited to participate anonymously in a research study that aims to investigate knowledge, attitudes, and practices of health students and practicing health professionals around counseling at-risk patients on health impacts of air pollution.

**Procedures:** As part of the study, you are being asked to complete a pre-survey, watch a short video on air pollution and health, and complete a post-survey. This research has been reviewed by the institutional IRB at the University of Minnesota and determined to be not human subjects research.

****Please be sure to follow the end-of-survey instructions to redeem your gift card!****

**Demographics**

Let's get started with a few questions about you

Q1 What degree are you pursuing/have you attained?

- MD/DO
- RN
- PA
- NP
- Other (fill in the blank) __________________________________________________

Q1A What is your current level of training?

- Medical Student
- Resident
- Fellow
- Post-training, 0-5 years
- Post-training, 5-10 years
- Post-training, 10+ years

Q1B What is your current level of training?

- RN Student
- Post-training, 0-5 years
- Post-training, 5-10 years
- Post-training, 10+ years

Q1C What is your current level of training?

- PA Student
- Resident/Fellow
- Post-training, 0-5 years
- Post-training, 5-10 years
- Post-training, 10+ years

Q1D What is your current level of training?

- NP Student
- Resident/Fellow
- Post-training, 0-5 years
- Post-training, 5-10 years
- Post-training, 10+ years

Q2 Which of the following best describes your gender identity?

- Cisgender male
- Cisgender female
- Non-binary
- Transgender male
- Transgender female
- Other (fill in the blank) __________________________________________________
- Prefer not to answer

Q3 What best describes your ethnic and/or racial background (select all that apply)?

- Hispanic, Latino, or Spanish
- Black or African American
- White/Caucasian
- Asian or Asian Indian
- Middle Eastern or Northern African
- Native American or Alaska Native
- Native Hawaiian or Pacific Islander
- Multiracial
- Some other race or ethnicity (fill in the blank) __________________________________________________
- Prefer not to answer

Q4 What is your specialty? (Indicate specialty not listed in "Other")

- Internal Medicine
- Pediatrics
- Family Medicine
- Other (fill in the blank) __________________________________________________
- N/A (student)

Q5 Which of the following location(s) best describe your current practice or training setting? (select all that apply)

- Urban
- Suburban
- Rural
- Tribal

Q6 Which of the following settings best describes the majority your training/practice?

- Non-patient facing
- Patient facing, outpatient
- Patient facing, inpatient
- Patient facing, both inpatient and outpatient
- Other (fill in the blank) __________________________________________________

Q7 What best describes your political leaning?

- Liberal
- Conservative
- No leaning/it varies
- Other (fill in the blank) __________________________________________________
- Prefer not to answer

**Pre-video Perceptions**

Welcome to the "pre-video" survey. We have 3 blocks of questions on your perceptions, clinical practice attitudes, and knowledge of the health impacts of air pollution. 
We start with a couple of questions on your **perceptions**:

Q8 For the following questions, please select the extent to which you agree or disagree with the statement:

|  | Strongly disagree | Disagree | Somewhat disagree | Neither agree nor disagree | Somewhat agree | Agree | Strongly agree |
| --- | --- | --- | --- | --- | --- | --- | --- |
| Air pollution negatively affects human health |  |  |  |  |  |  |  |
| Air quality in Minnesota is good enough that it does not have significant health impacts on Minnesotans |  |  |  |  |  |  |  |
| Air pollution impacts the health of patients that I currently care for in Minnesota |  |  |  |  |  |  |  |
| Air pollution has a significant impact on the health of Minnesotans who live in rural areas |  |  |  |  |  |  |  |
| Some Minnesotans are more negatively affected by air pollution than others |  |  |  |  |  |  |  |
| Climate change will contribute to worsening air quality over the coming decades |  |  |  |  |  |  |  |

Q9 For the following questions, please select the extent to which you agree or disagree with the statement:

|  | Strongly disagree | Disagree | Somewhat disagree | Neither agree nor disagree | Somewhat agree | Agree | Strongly agree |
| --- | --- | --- | --- | --- | --- | --- | --- |
| My patients are aware of the impacts of air pollution on health |  |  |  |  |  |  |  |
| My patients would want me to counsel them on the impacts of air pollution on their health |  |  |  |  |  |  |  |
| My patients would benefit from counseling on air pollution and health |  |  |  |  |  |  |  |
| I have wanted to counsel my patients on air pollution and health but have not done so due to lack of training/knowledge |  |  |  |  |  |  |  |

**Pre-Video Practice/Education**

Next, some questions on the health impacts of air pollution and your clinical practice:

Q10 For the following questions, please select how comfortable you feel with the following activities:

|  | Extremely uncomfortable | Somewhat uncomfortable | Neither comfortable nor uncomfortable | Somewhat comfortable | Extremely comfortable |
| --- | --- | --- | --- | --- | --- |
| Describing the health impacts of air pollution in an academic setting (e.g., when discussing a patient on rounds) |  |  |  |  |  |
| Identifying which patients are high-risk for health impacts secondary to air pollution in a clinical setting |  |  |  |  |  |
| Counseling patients on their personalized risk for negative health impacts secondary to air pollution |  |  |  |  |  |
| Counseling patients about health behaviors to protect themselves from the risks of air pollution |  |  |  |  |  |

Q11 For the following questions, please select the frequency with which the following situations occur:

|  | Daily | Weekly | Monthly | Yearly | Less than yearly | Never |
| --- | --- | --- | --- | --- | --- | --- |
| Patients ask me about how air pollution affects their health |  |  |  |  |  |  |
| I counsel patients on the health effects of air pollution |  |  |  |  |  |  |

Q12 During the course of your training, to what extent have you received training/education on

|  | A great deal | A lot | A moderate amount | A little | None at all |
| --- | --- | --- | --- | --- | --- |
| The health impacts of environmental factors such as air pollution or climate change |  |  |  |  |  |
| Identifying patients who are at risk of negative health impacts of environmental factors such as air pollution or climate change |  |  |  |  |  |
| Counseling at-risk patients on preventative behaviors to reduce the negative health impacts of environmental factors such as air pollution or climate change |  |  |  |  |  |

Q13 If you have NOT received formal training/education, but DO feel comfortable counseling patients, what is your current source of knowledge? (Please answer "N/A" if this does not apply to you)

**Pre-Video Knowledge**

Finally, a few short questions to evaluate your **knowledge** on the health impacts of air pollution before you watch the video:

Q14 Air pollution has been shown to be associated with the following health impacts:

|  | True | False | Unsure |
| --- | --- | --- | --- |
| Increased risk of stroke | - (Correct) |  |  |
| Increased risk of heart attack | - (Correct) |  |  |
| Increased risk of acute asthma exacerbation | - (Correct) |  |  |
| Increased risk of acute COPD exacerbation | - (Correct) |  |  |
| Increased risk of type 2 diabetes | - (Correct) |  |  |
| Increased risk of dementia | - (Correct) |  |  |

Q15 Approximately how many deaths per year does air pollution contribute to in Minnesota?

- <100
- 100 to 1,000
- 1,000 to 5,000 (Correct)
- >5,000

Q16 Of the following, which groups are the **most** sensitive to air pollution in Minnesota?

- Joggers and pet owners
- Children and those with pre-existing conditions such as lung and heart disease (Correct)
- Diabetics and those living in rural areas
- Unsure

Q17 Of the following, which groups are **most** likely to have high exposure to air pollution in Minnesota?

- Elderly people who live near parks
- People who commute a long distance in their air-conditioned car
- Communities of color (Correct)
- Unsure

Q18 Which group is **least** likely to have adaptive capacity (ability to protect themselves) regarding air pollution impacts?

- A family living in a newly-built apartment building
- A middle-aged person experiencing homelessness (Correct)
- A young couple living in Northern Minnesota
- Unsure

Q19 Which of the following Minnesotans is at **lowest** risk for experiencing health consequences related to air pollution?

- A 6 year old African American child with asthma who plays in her backyard next to I-94 highway
- A 64 year old uninsured Caucasian female with COPD who lives in rural Minnesota
- A healthy 54 year old Hispanic male who works at an insurance company and lives in the suburbs (Correct)
- Unsure

Q20 On days with poor air quality, what is the **best** way to limit harm from outdoor air pollution?

- Wear a fabric face mask when outdoors
- Do outdoor activities in the early afternoon, when air pollution is lowest
- Limit total time spent outdoors (Correct)
- Unsure

Q21 Do you know where to find information about the daily air quality in your area?

- Yes
- No

Q22 Do you know where to access didactic and clinical resources on the health impacts of air pollution?

- Yes
- No

Please proceed to the next section to watch our animated video on the health impacts of air pollution.

**Video**Please do not modify or recreate this video without permission. 
If you are unable to view this video in the survey, please copy and paste this link into another browser: [**https://youtu.be/xiY0AV3CjLE**](https://youtu.be/xiY0AV3CjLE)

**Post-Video Overview**

Now, we start the "post-video" survey. We have 2 blocks of questions. Let's start with an analysis of the video itself.

Q23 What surprised you most about the information that was presented in the video?

Q24 For the following questions, please select the extent to which you agree or disagree with the statement:

|  | Strongly agree | Somewhat agree | Neither agree nor disagree | Somewhat disagree | Strongly disagree |
| --- | --- | --- | --- | --- | --- |
| I learned useful information from this video |  |  |  |  |  |
| I enjoyed watching this video |  |  |  |  |  |
| I felt that the information presented in this video was easy to understand |  |  |  |  |  |
| I felt that this video was too simple or left out valuable information |  |  |  |  |  |
| I would be interested in viewing similar videos for other environmental hazards, such as heat-related illness, food and water pollution, or infectious disease (such as Lyme disease, Zika virus, or West Nile virus) |  |  |  |  |  |
| I would want my colleagues to watch this video |  |  |  |  |  |
| I would want my patients to watch this video |  |  |  |  |  |

Q25 For the following questions, please select how comfortable you feel with the following activities:

|  | Extremely uncomfortable | Somewhat uncomfortable | Neither comfortable nor uncomfortable | Somewhat comfortable | Extremely comfortable |
| --- | --- | --- | --- | --- | --- |
| Describing the health impacts of air pollution in an academic setting (e.g., when discussing a patient on rounds) |  |  |  |  |  |
| Identifying which patients are high-risk for health impacts secondary to air pollution in a clinical setting |  |  |  |  |  |
| Counseling patients on their personalized risk for negative health impacts secondary to air pollution |  |  |  |  |  |
| Counseling patients about health behaviors to protect themselves from the risks of air pollution |  |  |  |  |  |

Q26 For the following questions, please select the extent to which you agree or disagree with the statement:

|  | Strongly disagree | Disagree | Somewhat disagree | Neither agree nor disagree | Somewhat agree | Agree | Strongly agree |
| --- | --- | --- | --- | --- | --- | --- | --- |
| Air pollution negatively affects human health |  |  |  |  |  |  |  |
| Air quality in Minnesota is good enough that it does not have significant health impacts on Minnesotans |  |  |  |  |  |  |  |
| Air pollution impacts the health of patients that I currently care for in Minnesota |  |  |  |  |  |  |  |
| Air pollution has a significant impact on the health of Minnesotans who live in rural areas |  |  |  |  |  |  |  |
| Some Minnesotans are more negatively affected by air pollution than others |  |  |  |  |  |  |  |
| Climate change will contribute to worsening air quality over the coming decades |  |  |  |  |  |  |  |

Q27 For the following questions, please select the extent to which you agree or disagree with the statement:

|  | Strongly disagree | Disagree | Somewhat disagree | Neither agree nor disagree | Somewhat agree | Agree | Strongly agree |
| --- | --- | --- | --- | --- | --- | --- | --- |
| I would like continuing education on environmental health topics, such as the health effects of air pollution, to be offered. |  |  |  |  |  |  |  |

Q28 For the following questions, please select the extent to which you agree or disagree with the statement:

|  | Strongly disagree | Disagree | Somewhat disagree | Neither agree nor disagree | Somewhat agree | Agree | Strongly agree |
| --- | --- | --- | --- | --- | --- | --- | --- |
| I would like my training program to to give me clinical training on how to counsel patients on risks of air pollution and protective behaviors to mitigate these risks. |  |  |  |  |  |  |  |

Q28A In what format would you prefer to receive this clinical training? (select all that apply)

- Online recorded lectures to view at your own convenience
- Animated videos such as the one shown
- Reading material
- In-person lectures
- Standardized patient encounters
- Other (fill in the blank) __________________________________________________

Q29 As a result of watching this video, do you plan to identify and counsel patients who are at-risk for adverse health impacts of air pollution?

- Yes
- Maybe
- No

Q29A What prevents you from identifying and/or counseling at-risk patients? (select all that apply)

- I need more training
- There is limited time in a clinical encounter
- I don't think it's my role to counsel patients on the health impacts of air pollution
- I don't think my patients would find this information relevant
- I don't think that air pollution negatively impacts my patients
- Other (fill in the blank) __________________________________________________

Q30 Would you like your patients to be able to watch this video for educational purposes?

- Yes
- No

Q30A Please indicate which scenario(s) you think would be best for showing this video to at-risk patients (select all that apply):

- Via electronic medium (ex: MyChart message)
- In the waiting room
- During the clinical visit with the provider
- During the clinical visit with the RN/Medical Assistant
- Other (fill in the blank) __________________________________________________

Q31 If you are an educator, would you use this video as a teaching tool?

- Yes
- Maybe
- No
- N/A

**Post-Video Knowledge**

This is our last block of questions- a post-video survey of a few **knowledge** questions on the health impacts of air pollution.

Q32 Air pollution has been shown to be associated with the following health impacts:

|  | True | False | Unsure |
| --- | --- | --- | --- |
| Increased risk of stroke | - (Correct) |  |  |
| Increased risk of heart attack | - (Correct) |  |  |
| Increased risk of acute asthma exacerbation | - (Correct) |  |  |
| Increased risk of acute COPD exacerbation | - (Correct) |  |  |
| Increased risk of type 2 diabetes | - (Correct) |  |  |
| Increased risk of dementia | - (Correct) |  |  |

Q33 Approximately how many deaths per year does air pollution contribute to in Minnesota?

- <100
- 100 to 1,000
- 1,000 to 5,000 (Correct)
- >5,000

Q34 Of the following, which groups are the **most** sensitive to air pollution in Minnesota?

- Joggers and pet owners
- Children and those with pre-existing conditions such as lung and heart disease (Correct)
- Diabetics and those living in rural areas
- Unsure

Q35 Of the following, which groups are **most** likely to have high exposure to air pollution in Minnesota?

- Elderly people who live near parks
- People who commute a long distance in their air-conditioned car
- Communities of color (Correct)
- Unsure

Q36 Which group is **least** likely to have adaptive capacity (ability to protect themselves) regarding air pollution impacts?

- A family living in a newly-built apartment building
- A middle-aged person experiencing homelessness (Correct)
- A young couple living in Northern Minnesota
- Unsure

Q37 Which of the following Minnesotans is at **lowest** risk for experiencing health consequences related to air pollution?

- A 6 year old African American child with asthma who plays in her backyard next to I-94 highway
- A 64 year old uninsured Caucasian female with COPD who lives in rural Minnesota
- A healthy 54 year old Hispanic male who works at an insurance company and lives in the suburbs (Correct)
- Unsure

Q38 On days with poor air quality, what is the **best** way to limit harm from outdoor air pollution?

- Wear a fabric face mask when outdoors
- Do outdoor activities in the early afternoon, when air pollution is lowest
- Limit total time spent outdoors (Correct)
- Unsure

Q39 Do you know where to find information about the daily air quality in your area?

- Yes
- No

If you have any thoughts, comments, or constructive feedback on this video or survey, please let us know below!

________________________________________________________________

If you would like to collaborate with us on future projects, or would like to have access to this video to distribute to learners or patients, please reach out to the PI Dr. Laalitha Surapaneni at vsurapan@umn.edu
 
**Thank you so much for your participation in this survey! Please click the arrow in the lower right to submit your survey and *to access the link to receive your gift card*.

 *If you do not proceed past this page, you won't be able to receive your gift card!***
